# Magnolol Triggers Caspase-Mediated Apoptotic Cell Death in Human Oral Cancer Cells through JNK1/2 and p38 Pathways

**DOI:** 10.3390/biomedicines9101295

**Published:** 2021-09-22

**Authors:** Yi-Tzu Chen, Chiao-Wen Lin, Chun-Wen Su, Wei-En Yang, Chun-Yi Chuang, Shih-Chi Su, Ming-Ju Hsieh, Shun-Fa Yang

**Affiliations:** 1 School of Dentistry, Chung Shan Medical University, Taichung 402, Taiwan; chenyitzu0831@gmail.com; 2 Institute of Oral Sciences, Chung Shan Medical University, Taichung 402, Taiwan; cwlin@csmu.edu.tw; 3 Department of Dentistry, Chung Shan Medical University Hospital, Taichung 402, Taiwan; 4 Institute of Medicine, Chung Shan Medical University, Taichung 402, Taiwan; jeff11041986@gmail.com (C.-W.S.); weienyang@gmail.com (W.-E.Y.); 5 Department of Medical Research, Chung Shan Medical University Hospital, Taichung 402, Taiwan; 6 School of Medicine, Chung Shan Medical University, Taichung 402, Taiwan; cyi4602@gmail.com; 7 Department of Otolaryngology, Chung Shan Medical University Hospital, Taichung 402, Taiwan; 8 Whole-Genome Research Core Laboratory of Human Diseases, Chang Gung Memorial Hospital, Keelung 204, Taiwan; ssu1@cgmh.org.tw; 9 Department of Dermatology, Drug Hypersensitivity Clinical and Research Center, Chang Gung Memorial Hospital, Linkou 333, Taiwan; 10 Oral Cancer Research Center, Changhua Christian Hospital, Changhua 500, Taiwan; 11 Graduate Institute of Biomedical Sciences, China Medical University, Taichung 404, Taiwan

**Keywords:** oral cancer, magnolol, apoptosis, JNK1/2, p38

## Abstract

Magnolol is a natural compound extracted from Chinese herbal medicine and can induce apoptosis in numerous types of cancer cells. However, the molecular mechanisms of magnolol in oral cancer are still unclear. In this study, we investigated the anti-cancer effects and underlying mechanisms of magnolol in human oral cancer cell lines. Our results exhibited that magnolol inhibited the cell proliferation via inducing the sub-G1 phase and cell apoptosis of HSC-3 and SCC-9 cells. The human apoptosis array and Western blot assay showed that magnolol increased the expression of cleaved caspase-3 proteins and heme oxygenase-1 (HO-1). Moreover, we proved that magnolol induces apoptosis in oral cancer cell lines via the c-Jun N-terminal kinase (JNK)1/2 and p38 pathways. Overall, the current study supports the role for magnolol as a therapeutic approach for oral cancer through JNK1/2- and p38-mediated caspase activation.

## 1. Introduction

Oral cancer is the most common oral malignant tumor, accounting for 80–90% of all oral malignancies [1]. The causes of oral cancer are multifactorial and related to host immunity, metabolism, genetics, and exposure to chronic inflammation. These changes may be affected by oncogenes, carcinogens, and mutations caused by other chemicals, viruses, radiation, tobacco, and alcohol [2,3]. At present, the treatment of oral cancer is mainly based on surgery, and then it is decided to arrange postoperative radiotherapy, or even chemotherapy depending on the pathological staging and related risk factors after surgery [4]. However, some patients may be unable to undergo surgery due to the location of the tumor, the severity of the disease, or physical conditions, and concurrent chemo-radiotherapy may be the main treatment [4].

Magnolol (5,5′-diallyl-2,2′-dihydroxybiphenyl) is a structural isomer of honokiol; both are isolated from the traditional Chinese herbal medicine *Magnolia officinalis* [5]. Studies using purified magnolol have shown that it can have anti-inflammatory effects [6], anti-microbial activities [7], anti-oxidant effects [8], neuroprotection [9], cardiovascular protection [10], and anti-cancer properties [11]. Numerous previous in vitro and in vivo studies have demonstrated that magnolol has cancer chemo-preventive and inhibiting properties through different molecular mechanisms [11,12]. Jin et al. reported that magnolol could suppress cell migration and induced cell apoptosis via the inhibition of the nuclear factor-κB (NF-κB) signaling pathway in human multiple myeloma cells [13]. Su et al. observed that magnolol might, via extrinsic/intrinsic pathways, cause apoptosis in colorectal cancer [14]. Moreover, previous studies have confirmed that magnolol could inhibit cancer stemness and STAT3 signaling in oral cancer [15]. However, the effects of magnolol in oral cancer and its underlying mechanisms are still not well understood. In this study, we identified how magnolol treatment induced the apoptosis abilities of oral cancer cell lines and examined the underlying mechanisms.

## 2. Materials and Methods

### 2.1. Cell Culture

Human oral cancer cell lines HSC-3 were purchased from the Japanese Collection of Research Bioresources (Osaka, Japan). SCC-9 cell lines were purchased from the American Type Culture Collection (Manassas, VA, USA). Both HSC-3 and SCC-9 cell lines were cultured in Dulbecco’s modified Eagle’s medium and F-12 Ham’s medium as previously described [16].

### 2.2. Microculture Tetrazolium (MTT) Assay

The HSC-3 and SCC-9 oral cancer cells were seeded onto 24-well plates at a density of 8 × 10^4^ cells overnight and then treated with magnolol (M3445, Sigma-Aldrich, St. Louis, MO, USA) at different concentrations (0, 25, 50, 75, and 100 μM) for 24 h. Subsequently, the cells were incubated with MTT solution for 4 h at 37 °C as previously described [17].

### 2.3. Flow Cytometry Assay

To elucidate the cell cycle distribution, flow cytometric measurements of DNA content were determined as previously described [18]. Briefly, an 8 × 10^5^ /well of HSC-3 cells and 7 × 10^5^ /well of SCC-9 cells were cultured in 6 cm dishes, respectively, and treated with magnolol (0, 25, 50, 75, and 100 μM) for 24 h. Subsequently, cells were trypsinized, harvested, and fixed in 75% ethanol at −20 °C overnight. A PI/RNase staining buffer was added to cells at RT for 15 min in the dark, and then filtered and formulated for the PI labeling of DNA by flow cytometry (Beckman Coulter, Los Angeles, CA, USA).

### 2.4. Annexin V-FITC Staining Assay

The SCC-9 and HSC-3 cells were plated at a density of 5 × 10^5^ cells on 6 cm dishes and incubated with magnolol (0, 25, 50, 75, and 100 μM) for 24 h. After the removal of the old medium, cells were washed twice with PBS and then resuspended in a binding buffer. The solution was transferred to a new Eppendorf, and 5 µL of FITC Annexin V and 5 µL PI were added. Cells were then stained for 15 min at RT in the dark. The ratio of oral cancer cell apoptosis was evaluated by using the FITC Annexin V Apoptosis Detection Kit (BD Biosciences, San Jose, CA, USA) as previously described [19].

### 2.5. Western Blot Analysis

The preparation of SCC-9 and HSC-3 cell lysates for the Western blot analysis followed previously described procedures [18]. The Western blot analysis was performed with indicated primary and horseradish peroxidase-conjugated secondary antibodies. The primary antibodies were used as follows: pro-caspase-8 (#9746), pro-caspase-9 (#9502), cleaved caspase-8 (#9496), cleaved caspase-9 (#9505), cleaved caspase-3 (#9664), PARP (#9542), p-ERK (#4370), ERK (#9102), p-JNK (#4668), JNK (#9258), cIAP-1 (#7065, Cell Signaling Technology, MA, USA); pro-caspase-3 (610323), p38 (612168), p-p38 (612281, BD biosciences); HO-1 (ab68477), beta-actin (ab8226, Cambridge, MA, USA). Then, HRP-conjugated anti-mouse IgG (5450-0011, Seracare Life Sciences, Milford, MA, USA) or anti-rabbit IgG (5450-0010, Seracare Life Sciences, MA, USA) secondary antibodies were applied. All the blots were carried out with an enhanced chemiluminescence substrate solution (EMD Millipore Corporation, Burling, MA, USA) to produce images. The band intensities were quantified by NIH ImageJ analysis software.

### 2.6. Human Apoptosis Array

To elaborate the underlying mechanism of apoptosis induced by Magnolol, a human apoptosis array was performed. HSC-3 cells (6 × 10^5^) were seeded in 6 cm dishes. After treatment with Magnolol (100 μM) for 24 h, cell lysates (400 µg) were collected using a Human Apoptosis Array Kit (ARY009) (R&D Systems, Minneapolis, MN, USA) according to the recommended protocol. The results detected the relative expression levels of 35 apoptosis-related proteins and provided 2 biological replicates normalized with internal controls.

### 2.7. Statistical Analysis

Data are shown as the means ± standard deviations. Differences between the control and magnolol-treated groups were evaluated using ANOVA analysis with Tukey’s posteriori comparison. A difference was considered statistically significant at a *p* value of < 0.05.

## 3. Results

### 3.1. Magnolol Inhibited Cell Viability in Oral Cancer Cell Lines

**Figure 1:**
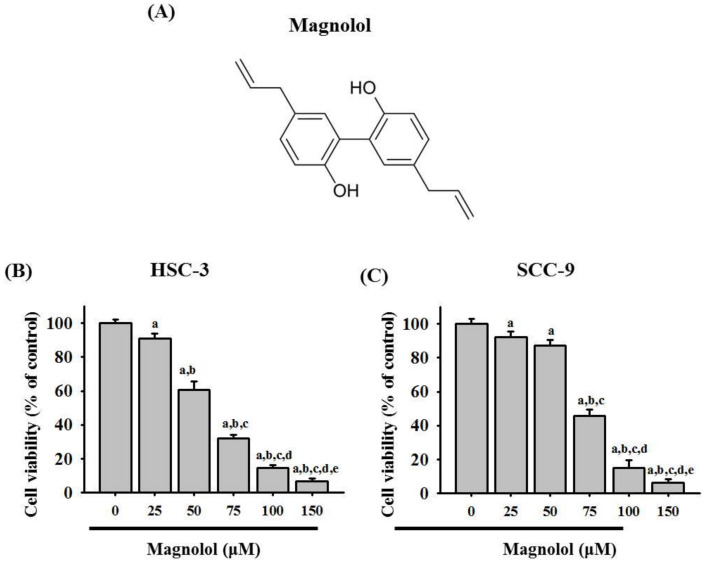
Magnolol inhibited cell viability in HSC-3 and SCC-9 oral cancer cell lines. (**A**) The structure of magnolol. (**B**,**C**) After treatment with magnolol (0, 25, 50, 75, 100, and 150 μM) for 24 h, the cell viability of HSC-3 and SCC-9 cells was detected by using an MTT assay. The data represent the mean ± SD of three independent experiments. ^a^ Significantly different, *p* < 0.05, when compared to control. ^b^ Significantly different, *p* < 0.05, when compared to 25 μM. ^c^ Significantly different, *p* < 0.05, when compared to 50 μM. ^d^ Significantly different, *p* < 0.05, when compared to 75 μM. ^e^ Significantly different, *p* < 0.05, when compared to 100 μM.

To investigate the effect of magnolol on the oral cancer cell viability, SCC-9 and HSC-3 cells were treated with magnolol at concentrations of 0, 25, 50, 75, 100, and 150 μM for 24 h (Figure 1). MTT assay revealed that magnolol inhibited the cell viability in HSC-3 and SCC-9 cell lines (Figure 1B,C).

### 3.2. Magnolol-Induced Apoptosis in Human Oral Cancer Cell Lines

**Figure 2:**
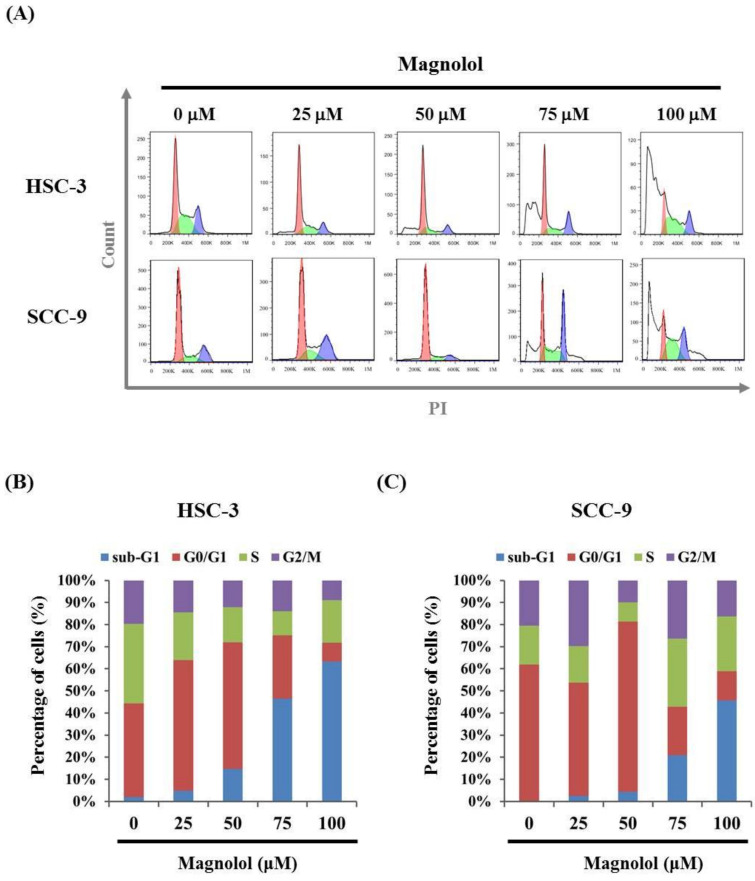
Effects of magnolol on the cell-cycle phase distribution of HSC-3 and SCC-9 cells. (**A**) After treatment with magnolol (0, 25, 50, 75, 100, and 150 μM) for 24 h, the cell-cycle phase distribution in the HSC-3 and SCC-9 cells was evaluated by flow cytometry assay, as described in the Materials and Methods section. Quantitative cell-cycle phase distribution indicated that sub-G1 arrest was induced by magnolol on (**B**) HSC-3 cells and (**C**) SCC-9 cells in a dose-dependent manner.

**Figure 3:**
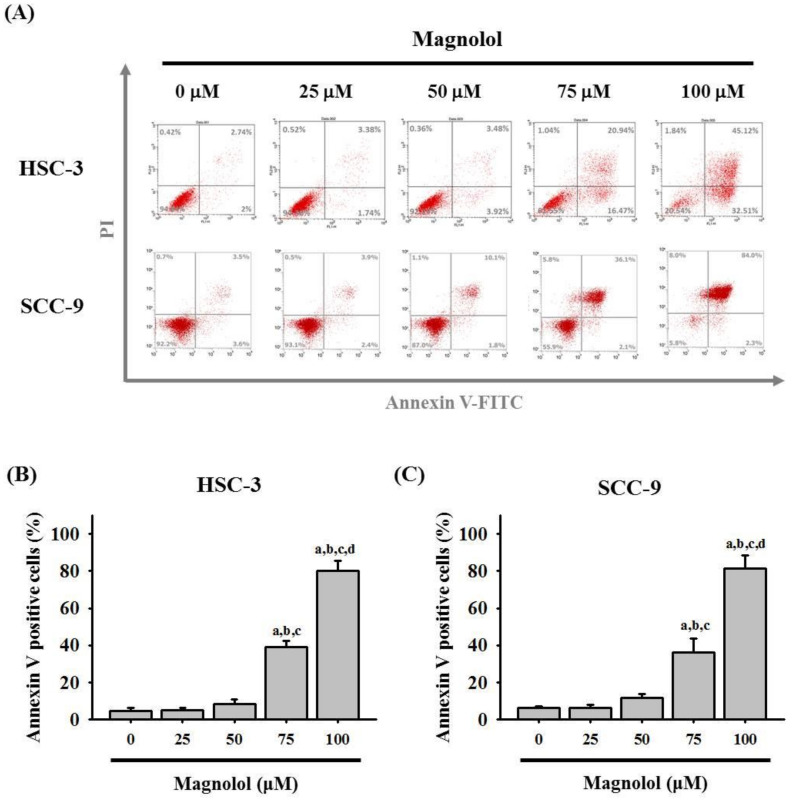
Magnolol induced HSC-3 and SCC-9 cell apoptosis. (**A**) After treatment with magnolol (0, 25, 50, 75, and 100 μM) for 24 h, HSC-3 and SCC-9 cells were treated with Annexin V-FITC and PI and analyzed through flow cytometry, as described in the Materials and Methods section. Quantitative Annexin V positive cells were induced by magnolol on (**B**) HSC-3 cells and (**C**) SCC-9 cells in a dose-dependent manner. The data represent the mean ± SD of three independent experiments. ^a^ Significantly different, *p* < 0.05, when compared to control. ^b^ Significantly different, *p* < 0.05, when compared to 25 μM. ^c^ Significantly different, *p* < 0.05, when compared to 50 μM. ^d^ Significantly different, *p* < 0.05, when compared to 75 μM.

As shown in Figure 2A–C, after treatment with magnolol for 24 h, the percentages in the sub-G1 phase of HSC-3 and SCC-9 cells significantly increased in a dose-dependent manner. Moreover, the apoptotic cells significantly increased in a dose-dependent manner in the Annexin V- FITC/PI apoptosis assay (Figure 3A–C). These results demonstrated that magnolol may induce apoptotic cell death in oral cancer cells.

### 3.3. Magnolol Triggers Caspase-Mediated Apoptosis in Oral Cancer

**Figure 4:**
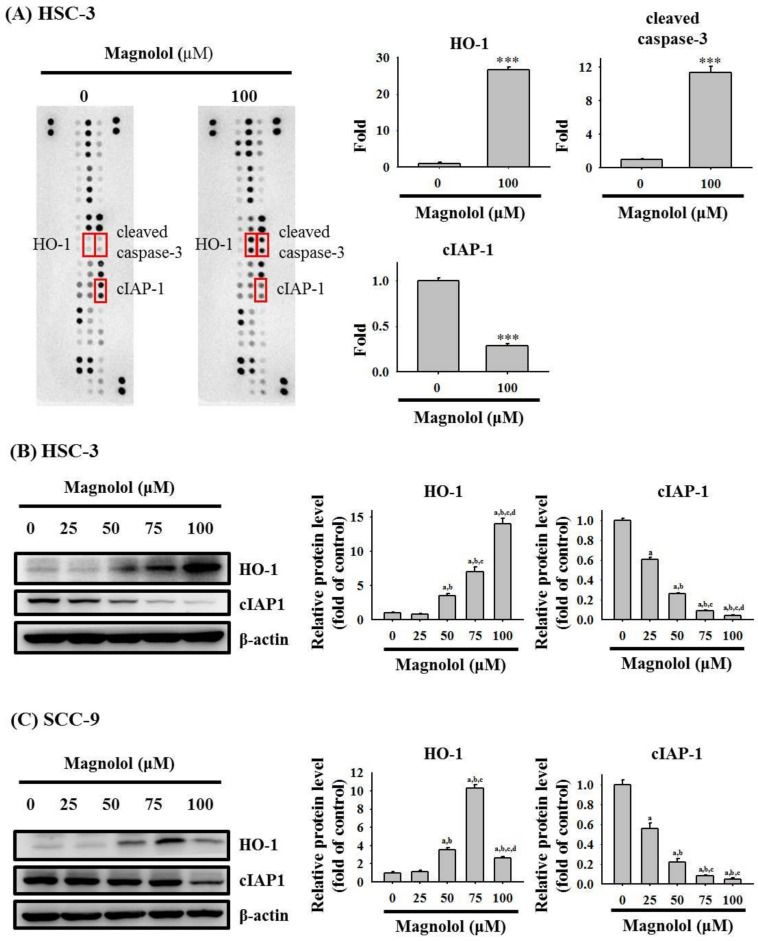
Screening of apoptosis-related proteins modulated by magnolol in oral cancer HSC-3 cells. (**A**) Whole cell lysate from HSC-3 cells was treated with or without magnolol (100 μM) for 24 h via apoptotic protein array analysis. *** *p* < 0.005, compared with control group. (**B**,**C**) Protein levels of HO-1 and cIAP-1 proteins in the (**B**) HSC-3 cells and (**C**) SCC-9 cells were measured through Western blot assay. The data represent the mean ± SD of three independent experiments. ^a^ Significantly different, *p* < 0.05, when compared to control. ^b^ Significantly different, *p* < 0.05, when compared to 25 μM. ^c^ Significantly different, *p* < 0.05, when compared to 50 μM. ^d^ Significantly different, *p* < 0.05, when compared to 75 μM.

**Figure 5:**
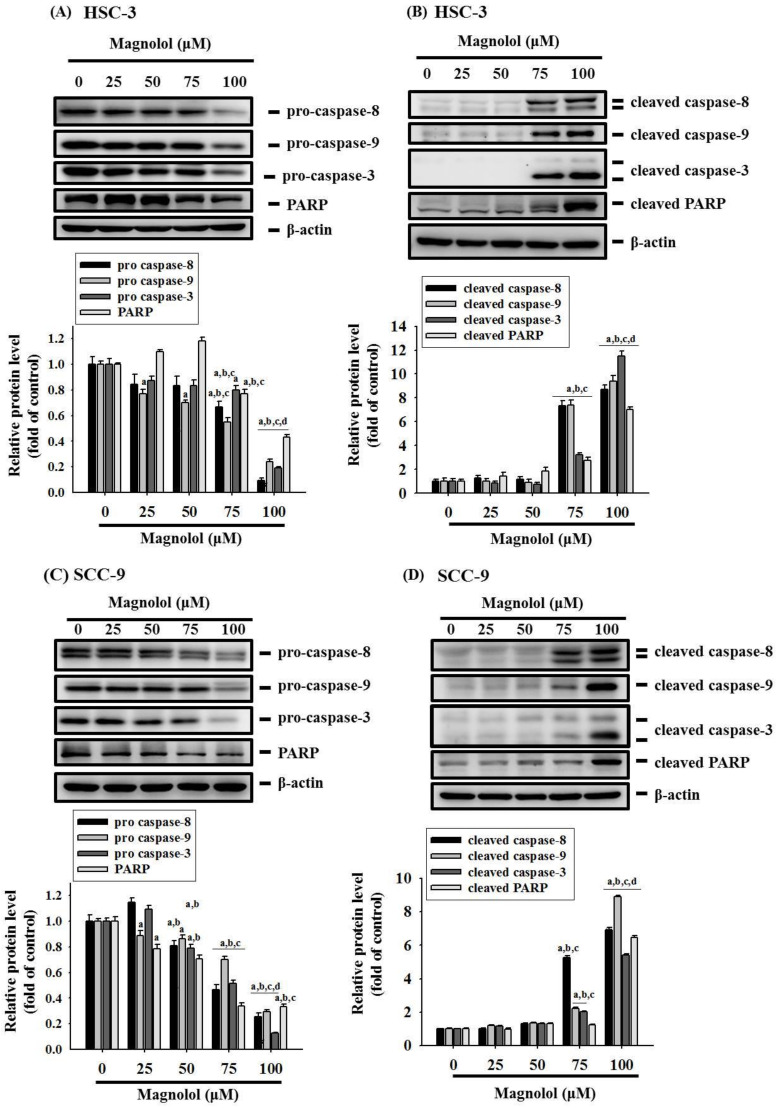
Magnolol triggers caspase-mediated apoptosis in HSC-3 and SCC-9 cells. (**A**,**B**) HSC-3 cells and (**C**,**D**) SCC-9 cells were treated with magnolol (0, 25, 50, 75, and 100 μM) for 24 h, and the expression levels of pro-form and cleaved-form caspase-3, -8, and -9, and PARP, were detected by Western blot analysis. The data represent the mean ± SD of three independent experiments. ^a^ Significantly different, *p* < 0.05, when compared to control. ^b^ Significantly different, *p* < 0.05, when compared to 25 μM. ^c^ Significantly different, *p* < 0.05, when compared to 50 μM. ^d^ Significantly different, *p* < 0.05, when compared to 75 μM.

To classify the mechanism of magnolol-induced apoptosis in oral cancer cells, the Proteome Profiler Human Apoptosis Array was employed to determine apoptosis-related protein. As shown in Figure 4A, obvious increases of the cleaved caspase 3 proteins and heme oxygenase-1 (HO-1) expression and decreases in cIAP-1 proteins in HSC-3 cells were observed after treatment with 100 μM magnolol for 24 h. We also conducted a Western blot assay to validate the findings of the apoptosis array and found that magnolol significantly increased the HO-1 expression and repressed the cIAP-1 level in HSC-3 cells (Figure 4B) and SCC-9 cells (Figure 4C). Moreover, after treatment with various concentrations of magnolol in HSC-3 cells for 24 h, magnolol induced the degradation of pro-caspase-8, -9, and -3, as well as poly (ADP-ribose) polymerase (PARP), which generated active forms of caspase-8, -9, and -3, and PARP (Figure 5A,B). Similar results to those of HSC-3 cells were also observed in magnolol-treated SCC-9 cells (Figure 5C,D).

### 3.4. Activation of the MAPK Signaling Pathway by Magnolol in Oral Cancer Cell

**Figure 6:**
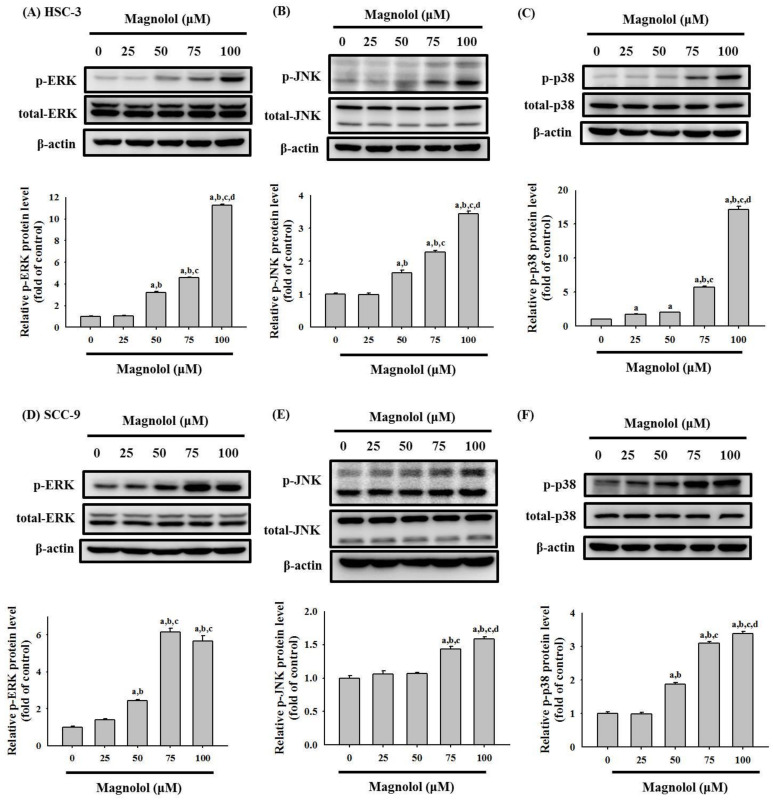
Magnolol activated the MAPK pathway in the HSC-3 and SCC-9 cell lines. (**A**–**C**) HSC-3 cells and (**D**–**F**) SCC-9 cells were treated with magnolol (0, 25, 50, 75, and 100 μM) for 6 h. The phosphorylated levels of (**A**,**D**) ERK1/2, (**B**,**E**) JNK1/2, and (**C**,**F**) p38 were assessed using Western blot analysis, as described in the Materials and Methods section. The data represent the mean ± SD of three independent experiments. ^a^ Significantly different, *p* < 0.05, when compared to control. ^b^ Significantly different, *p* < 0.05, when compared to 25 μM. ^c^ Significantly different, *p* < 0.05, when compared to 50 μM. ^d^ Significantly different, *p* < 0.05, when compared to 75 μM.

**Figure 7:**
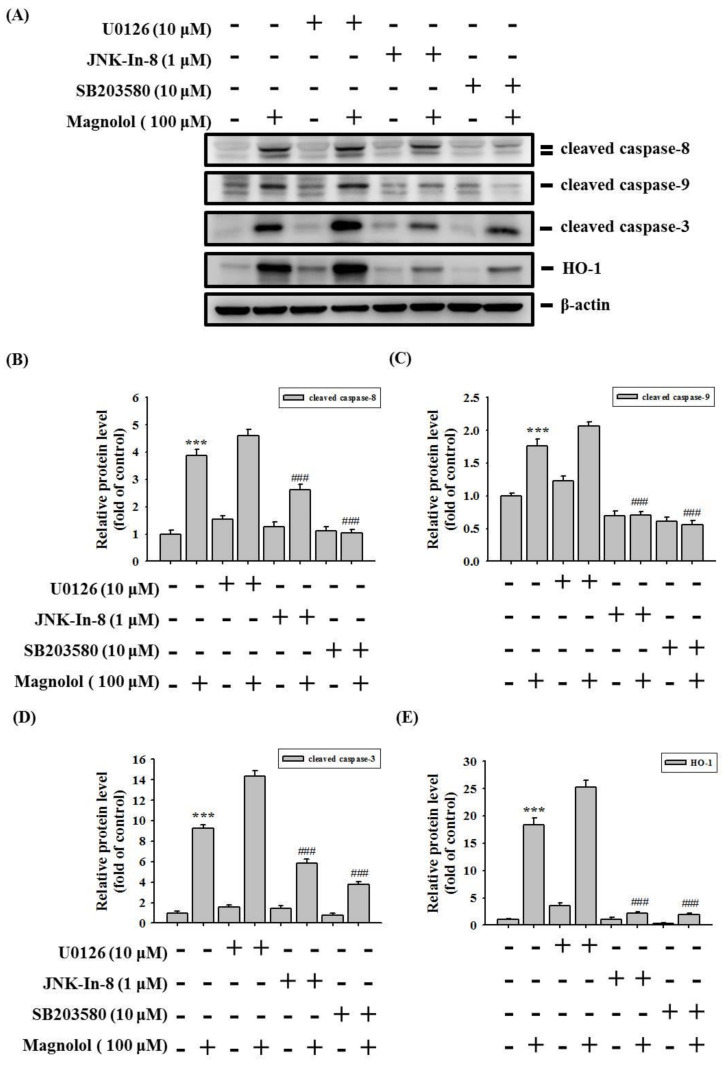
Magnolol induced caspase activation and HO-1 expression through JNK1/2 and p38 pathways in the HSC-3 cell line. (**A**) HSC-3 cells were pretreated with ERK1/2 inhibitor U0126 (10 μM), JNK1/2 inhibitor JNK-IN-8 (1 μM), or p38 inhibitor SB203580 (10 μM) for 1 h and then treated with 100 μM magnolol (100 μM) for 24 h. The expressions of cleaved caspase-9, -8, and -3, and HO-1, were detected using Western blot analysis, as described in the Materials and Methods section. (**B**–**E**) Quantitative results of cleaved caspase-9, -8, and -3, and HO-1 protein levels, were adjusted for β-actin. The data represent the mean ± SD of three independent experiments. *** *p* < 0.005 compared with the control group. ^###^ *p* < 0.05 compared with the magnolol alone treatment group.

We examined whether magnolol can induce the activation of the ERK1/2, JNK1/2, and p38 pathways in HSC-3 and SCC-9 cells. As shown in Figure 6A–F, magnolol significantly increased the phosphorylation of ERK1/2, JNK1/2 and p38 in a concentration-dependent manner in both HSC-3 and SCC-9 oral cancer cell lines. We further used the specific ERK1/2 inhibitor (U0126), specific JNK1/2 inhibitor (JNK-IN-8), and specific p38 inhibitor (SB203580), to verify the involvement of the MAPK pathway in magnolol-induced apoptosis. As shown in Figure 7A–E, pretreatment with the JNK1/2 inhibitor (JNK-IN-8) and p38 inhibitor (SB203580) effectively reversed the expression of cleavage caspase-8, -9, and -3, and HO-1, compared with the magnolol-only-treated HSC-3 oral cancer cells. Our results suggested that the JNK1/2 and p38 pathways play a critical role in magnolol-induced oral cancer apoptosis in HSC-3 cells.

## 4. Discussion

Our present results indicate that magnolol inhibited the proliferation of HSC-3 and SCC-9 cells. Flow cytometry results indicated that apoptosis occurred in magnolol-treated HSC-3 and SCC-9 cells. The human apoptosis array and Western blot assay showed that in HSC-3 cells, the expression of HO-1 and cleaved caspase-3 increased, while cIAP-1 expression decreased after magnolol treatment. Finally, we proved that magnolol induces apoptosis in oral cancer cell lines via the JNK- and p38-mediated MAPK pathway.

Heme oxygenase-1 (HO-1) plays a vital role in anti-inflammatory, anti-oxidant, and anti-apoptotic properties [20,21,22]. Nitti et al. revealed that HO-1 can induce resistance to therapies, leading to poor outcome in cancer cells [22]. A similar finding was that the anti-cancer effects of isoliquiritigenin 2′-methyl ether [23] and mollugin [24] may be involved in HO-1 upregulation via the MAP kinase pathway. Moreover, significantly higher HO-1 levels were found in oral cancer tissue, and a higher HO-1 expression was also associated with oral cancer lymph node metastasis [25]. In this study, we observed that magnolol could induce HO-1 upregulation and induce apoptosis in oral cancer cells.

c-IAP1, a member of the IAP family, could affect canonical and non-canonical NF-κB signaling, which could mediate apoptotic effects [26]. In this study, our results revealed that magnolol significantly repressed the cIAP-1 level in oral cancer cells. Pang et al. reported that ursodeoxycholic acid induces apoptosis in HSC-3 cancer cells through caspase activation, which inhibits the translocation of NF-κB and the downregulation of c-IAP1 [27]. Thus, whether NF-κB signaling is involved in the magnolol-medicated downregulation of c-IAP1 in oral cancer, warrants further investigation.

Our results indicate that magnolol induces oral cancer cell apoptosis by increasing the cleavage of caspase and PARP. Other studies observed the release of caspase-8, -9, and -3, as well as PARP cleavage, after treatment with magnolia species and led to DNA fragmentation [28,29,30]. Chen et al. reported that magnolol enhanced the therapeutic efficacy of regorafenib though the induction of apoptosis by inhibiting myeloid cell leukemia 1 (Mcl-1) and VEGFA expression in hepatocellular carcinoma cells [31]. Moreover, Woo et al. also demonstrated that magnolol sensitizes TRAIL-induced apoptotic cell death via the downregulation of Mcl-1 expression in A549 lung cancer cells [32]. Thus, further study to investigate the involvement of the magnolol-induced downregulation of Mcl-1 in oral cancer is required.

In our study, the results showed that magnolol significantly increased the phosphorylation of ERK1/2, JNK1/2 and p38 in oral cancer cell lines (Figure 6). However, pretreatment with a specific ERK1/2 inhibitor (U0126) did not reverse the magnolol-induced cleavage caspase expression. Previous reports revealed that the ERK1/2 signal pathway involved in isorhamnetin-induced paraptopic cell death in oral cancer cell lines [33]. Chen et al. demonstrated that ERK1/2 activations were positively associated with intracellular reactive oxygen species levels [33]. Thus, the role of magnolol-induced ERK1/2 activation in oral cancer requires further study. Moreover, several studies have revealed that JNK1/2 plays an anti-tumor role in oral cancer and emphasizes its positive role in apoptosis [34,35,36]. Numerous studies have also reported that magnolol inhibits cancer progression [14] or induces apoptosis [13,37] by regulating multiple cellular signaling pathways. Su et al. reported that magnolol induces colorectal cancer apoptosis through both extrinsic/intrinsic pathways via PKCδ and NF-κB signaling [14]. Moreover, in multiple myeloma, magnolol induced cell apoptosis and inhibited cell migration by upregulating miR-129 and inhibiting NF-κB pathway activation [13]. Chen et al. demonstrated that magnolol induced apoptosis by activating the ERK1/2 and p38 pathways in esophagus cancer KYSE150 cells [38]. Our study is the first study to reveal that magnolol induces apoptosis in oral cancer cell lines via the JNK1/2 and p38 pathways.

## 5. Conclusions

In conclusion, magnolol induces apoptosis in oral cancer cell lines via the JNK1/2- and p38-mediated MAPK pathways. Our findings indicated that magnolol is a potential therapeutic agent for oral cancer. The therapeutic potential of magnolol in oral cancer treatment warrants evaluation in future research.

## Data Availability

The data presented in this study are available upon request from the corresponding author.
